# PIP5k1**β** controls bone homeostasis through modulating both osteoclast and osteoblast differentiation

**DOI:** 10.1093/jmcb/mjz028

**Published:** 2019-04-16

**Authors:** Xiaoying Zhao, Penglei Cui, Guoli Hu, Chuandong Wang, Lei Jiang, Jingyu Zhao, Jiake Xu, Xiaoling Zhang

**Affiliations:** 1 Department of Orthopedic Surgery, Xinhua Hospital, School of Medicine, Shanghai Jiao Tong University, Shanghai 200092, China; 2 The Key Laboratory of Stem Cell Biology, Shanghai Institutes for Biological Sciences, Chinese Academy of Sciences, Shanghai 200025, China; 3 School of Pathology and Laboratory Medicine, University of Western Australia, Perth, Western Australia, 6009, Australia; 4 Key Laboratory of Tibetan Medicine Research, Northwest Plateau Institute of Biology, Chinese Academy of Sciences, Xining 810001, China

**Keywords:** PIP5k1β, osteoclast differentiation, osteoblast differentiation, proliferation, migration, NFATC1

## Abstract

PIP5k1**β** is crucial to the generation of phosphotidylinosotol (4, 5)P_2_. PIP5k1**β** participates in numerous cellular activities, such as B cell and platelet activation, cell phagocytosis and endocytosis, cell apoptosis, and cytoskeletal organization. In the present work, we aimed to examine the function of PIP5k1**β** in osteoclastogenesis and osteogenesis to provide promising strategies for osteoporosis prevention and treatment. We discovered that PIP5k1**β** deletion in mice resulted in obvious bone loss and that PIP5k1**β** was highly expressed during both osteoclast and osteoblast differentiation. Deletion of the gene was found to enhance the proliferation and migration of bone marrow-derived macrophage-like cells to promote osteoclast differentiation. PIP5k1**β**^−/−^ osteoclasts exhibited normal cytoskeleton architecture but stronger resorption activity. PIP5k1**β** deficiency also promoted activation of mitogen-activated kinase and Akt signaling, enhanced TRAF6 and c-Fos expression, facilitated the expression and nuclear translocation of NFATC1, and upregulated Grb2 expression, thereby accelerating osteoclast differentiation and function. Finally, PIP5k1**β** enhanced osteoblast differentiation by upregulating master gene expression through triggering smad1/5/8 signaling. Therefore, PIP5k1**β** modulates bone homeostasis and remodeling.

## Introduction

Bone remodeling depends on the dynamic balance and precise coordination of bone resorption and subsequent bone formation, which are driven by osteoclast and osteoblast activation, respectively. Disturbances of this delicate balance lead to skeletal diseases, such as osteopenia, osteoporosis, and osteopetrosis (Boyle et al., 2003). Bone-resorbing osteoclasts are multinucleated cells derived from monocyte-macrophage precursors that originated from hematopoietic stem cells, whereas bone-forming osteoblasts are derived from mesenchymal stem cells (MSCs) (Dennis et al., 1999; Pittenger et al., 1999; Teitelbaum, 2000; Boyle et al., 2003). Osteoblast lineage cells produce receptor activator of nuclear factor kappa-B ligand (RANKL), which stimulates their receptor activator of nuclear factor kappa-B (RANK) receptors on osteoclast precursors, resulting in osteoclast differentiation by activation of downstream signaling pathways such as the transcription factors nuclear factor-κB (NF-κB), c-Fos, and nuclear factor of activated T cells c1 (NFATC1) (Teitelbaum and Ross, 2003). To initiate bone resorption, osteoclasts need to successfully assemble a typical adhesion structure that is called an actin ring or sealing zone, which consists of a belt of densely packed podosomes interlinked by an acto-myosin network (Georgess et al., 2014; Touaitahuata et al., 2014).

Exacerbated by general population aging, osteoporosis has become a dominating health problem around the world (Sambrook and Cooper, 2006). Various conditions can induce osteoporosis, such as bone metastasis, disability, and inflammation. Furthermore, in postmenopausal women, estrogen deficiency gives rise to excessive bone loss (Touaitahuata et al., 2016). Despite recent advances in bone biology, the precise molecular mechanisms responsible for pathological osteoporosis remain elusive. Therefore, clarifying the molecular mechanisms and novel molecules involved in the maintenance of bone homeostasis is critical for a more comprehensive understanding of skeletal health and development of novel therapeutics against various bone disorders.

Lipid kinases and their phosphoinositide (PI) products perform essential functions in secretory vesicle trafficking. PI contributes to numerous basic biological processes, such as chemotaxis, intercellular trafficking, polarity formation, and cytokinesis (Takenawa and Itoh, 2001). PIs are essential not only as membrane components in eukaryotes and as precursors of second messengers like IP3 and PIP3 but also act as specialized membrane docking sites for effectors of diverse signaling cascades (Takenawa and Itoh, 2001). Accumulating evidence indicates that PIs and PI-interacting proteins such as Rho, Arf, and Rab small GTPases serve as modulators of osteoclast differentiation (Chellaiah, 2006; Ory et al., 2008). PIs, especially PIP2, PIP3, and IP3, have been reported to serve various essential roles during osteoclast differentiation and function as well as in maintaining bone homeostasis. PIP5k1β is a member of the Type 1 phosphatidylinositol 4-phosphate 5-kinases (PIP5k1s; α, β, and γ), which are a family of isoenzymes producing phosphatidylinositol 4,5-bisphosphate [PI(4,5)P_2_] using phosphatidylinositol 4-phosphate as substrate (Ishihara et al., 1996; Ishihara et al., 1998; De Matteis and Godi, 2004; Oude Weernink et al., 2004; Kanaho et al., 2007; van den Bout and Divecha, 2009). PIP5k1s have been reported to be involved in a wide range of cellular functions, such as enzymatic generation of PIP2, which is a critical regulator of cell adhesion formation, actin dynamics, and membrane trafficking, and as a cell signaling modulation factor involved in cytoskeleton assembly, exocytosis, endocytosis, and cell apoptosis (Mao and Yin, 2007; van den Bout and Divecha, 2009). PIP5k1γ, which is a major PtdIns(4,5)P_2_-synthesising enzyme in the rodent brain with three splicing variants, is elegantly involved in neurons and neuroendocrine cells. Hara et al. (2013) reported that PIP5k1γ, particularly PIP5k1γ_i2, performs essential roles in neuronal migration, possibly through recruitment of adhesion components such as talin and focal adhesion kinase to the plasma membrane. In murine megakaryocytes, PIP5k1γ defect results in plasma membrane blebbing accompanied by a decreased connection of the membrane to the cytoskeleton possibly through a pathway involving talin (Wang et al., 2008b). Moreover, PIP5k1γ_i2 displays a unique regulatory and targeting mechanism through its C-terminal 26 amino acids. In neurons, PIP5k1γ_i2 modulates the clathrin-mediated endocytosis of synaptic vesicles at presynapses and α-amino-3-hydroxy-5-methyl-4-isoxazolepropionate-type glutamate receptors during long-term depression at postsynapses via association with adaptor protein complex 2 (Unoki et al., 2012). In non-neuronal cells, PIP5k1γ_i2 also regulates the formation of focal adhesions through interaction with talin (Di Paolo et al., 2002; Ling et al., 2002; Sun et al., 2007). Furthermore, targeted disruption of PIP5k1γ leads to widespread developmental and cellular defects. PIP5k1γ-null embryos have myocardial developmental defects including impaired intracellular junctions resulting in heart failure and extensive lethality at embryonic day 11.5 as well as impaired PIP2 production, adhesion junction formation, and neuronal cell migration that lead to neural tube closure defects (Wang et al., 2007). PIP5k1α was reported to selectively modulate apical endocytosis in polarized renal epithelial cells and to regulate invadopodia formation and extracellular matrix degradation in human breast cancer cells by localized production of PI(4,5)P_2_ (Szalinski et al., 2013). The function of PIP5k1β on the other hand has not been well studied, and the roles of PIP5k1s in maintaining bone homeostasis have been poorly characterized. One study indicated that PIP5k1γ deficiency or overexpression delayed osteoclast differentiation and an excess of PIP5k1γ disrupted the osteoclast cytoskeleton in a talin-independent way (Zhu et al., 2013).

In the present study, we found that PIP5k1β was highly expressed during RANKL-induced osteoclast differentiation and PIP5k1β deletion in mice resulted in bone loss. To further understand the role of PIP5k1β in bone homeostasis, we investigated the role of PIP5k1β in osteoclast and osteoblast differentiation by gain- and loss-of-function studies *in vitro*. We found that PIP5k1β can repress the proliferation and migration of bone marrow-derived macrophage-like cells (BMMs), inhibiting osteoclast differentiation. Furthermore, PIP5k1β deletion promoted the activation of mitogen-activated kinase (MAPK) and Akt signaling cascades and enhanced expression of TRAF6 and c-Fos to facilitate NFATC1 expression and nuclear translocation, thereby accelerating osteoclast differentiation and function. PIP5k1β deficiency also resulted in upregulation of growth factor receptor-bound protein 2 (Grb2) expression during osteoclast differentiation. In osteoblasts, PIP5k1β was found to enhance osteoblasts differentiation through activation of smad1/5/8. Thus, PIP5k1β can regulate bone mass and bone remodeling.

**Figure 1:**
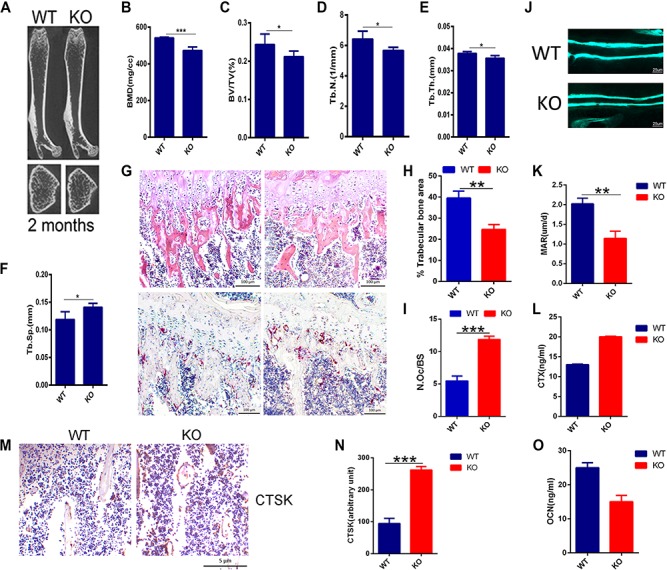
PIP5k1β-deficient mice exhibit an osteoporotic bone phenotype. (**A**) Representative micro-CT images of femurs from WT or PIP5k1β knockout 2-month-old male mice. (**B**–**F**) BMD, BV/TV, Tb.N., Tb.Th., and Tb.Sp. were assessed from the micro-CT measurements (*n* = 10). (**G**) The H&E (upper) and TRAP staining (lower) of histological sections of proximal tibiae. Scale bar, 100 μm. (**H** and **I**) Analysis of trabecular bone area and quantification of osteoclast number/bone surface (N.Oc/BS) in **G**. *n* = 10. (**J** and **K**) Representative images of new bone formation (**J**) and quantification of MAR (**K**) as assayed by calcein double labeling (*n* = 6). (**L**) Serum concentration of CTX-1 in WT or PIP5k1β knockout mice (*n* = 6). (**M** and **N**) The expression of CTSK in WT or PIP5k1β knockout mice shown by immunohistochemistry (**M**) and the staining density were quantified by ImageJ (**N**). *n* = 6 per group. (**O**) Serum concentration of OCN in WT or PIP5k1β knockout mice (*n* = 6). Data represent mean ± SD of three independent experiments. *P < 0.05; **P < 0.01; ***P < 0.001.

**Figure 2:**
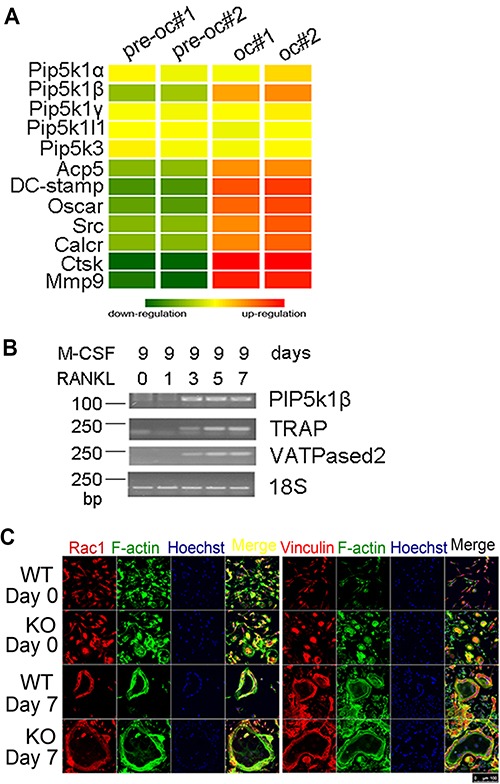
PIP5k1β expression was increased during RANKL-induced osteoclast differentiation and PIP5k1β-deficient osteoclasts exhibit normal morphology. (**A**) cDNA from pre-osteoclasts and mature osteoclasts were subjected to microarray analysis and the expression levels of PIP5k family genes and osteoclast master genes were determined. The heat map is ordered by degree of differential expression of the indicated genes between pre-osteoclasts and mature osteoclasts. (**B**) RT-PCR detection of the expression levels of PIP5k1β, TRAP, and v-ATPase-d2 during osteoclastogenesis, with 18S RNA as a control. (**C**) BMMs from WT or PIP5k1β^−/−^ mice underwent osteoclastogenesis by stimulation with 20 ng/ml M-CSF and 75 ng/ml RANKL for 7 days. The actin ring formation, Vinculin, and Rac1 expression and colocalization with F-actin were assessed by classical confocal microscopy. F-actin was stained with phalloidin (green) and nuclei were stained with Hoechst 3342. All the data were confirmed by three independent experiments.

**Figure 3:**
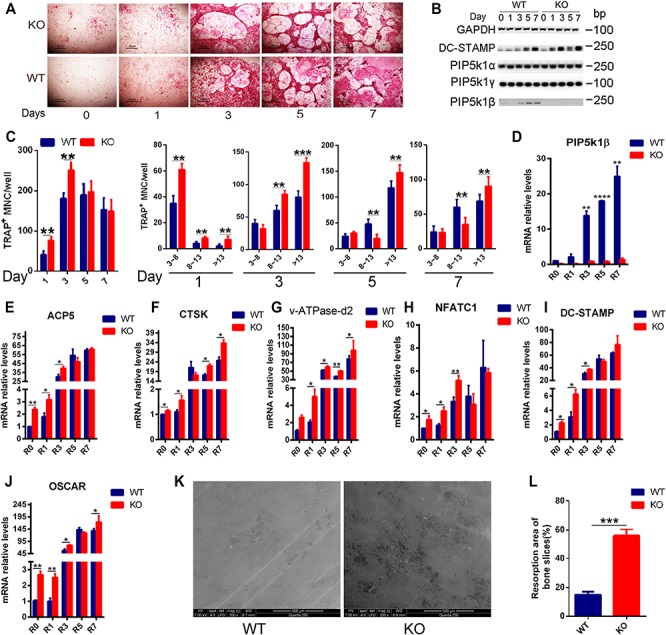
Deficiency of PIP5k1β enhanced osteoclast differentiation and bone resorption. (**A** and **C**) BMMs from WT or PIP5k1β^−/−^ mice underwent osteoclastogenesis by stimulation with 20 ng/ml M-CSF and 75 ng/ml RANKL for the indicated number of days. The formation of osteoclasts was detected by TRAP staining (**A**) and the number of osteoclasts was counted (**C**) (*n* = 3). (**B**, **D**–**J**) PIP5k1β, PIP5k1α, PIP5k1γ, and osteoclast-specific (ACP5, CTSK, v-ATPase-d2, NFATC1, DC-STAMP, and OSCAR) gene expression was analyzed by RT-PCR (**B**) and quantitative real-time PCR (Q-PCR) (**D**–**J**), normalized to the expression of GAPDH. All experiments were performed at least three times. (**K**) WT (left) or PIP5k1β^−/−^ (right) BMMs were cultured on bovine bone slices with 20 ng/ml M-CSF and 75 ng/ml RANKL for 8 days, with Pit formation detected by SEM. (**L**) Pit formation rate was measured by Image J and presented graphically. All the experiments were repeated three times. Data represent mean ± SD of three independent tests. *P < 0.05; **P < 0.01; ***P < 0.001; ****P < 0.0001 vs. WT.

## Results

### PIP5k1β-deficient mice show an osteoporosis bone phenotype

It has been demonstrated that PIP5k1γ serves an essential role in modulating osteoclast differentiation and that it must be expressed at a precise level to maintain normal osteoclast differentiation (Zhu et al., 2013).To determine the role of PIP5k1β in the skeleton, we evaluated the mutant mouse strain with piggyback (PB) transposition system-induced mutation in the *PIP5k1β* gene. Homozygous PIP5k1β mutants were born healthy at the predicted Mendelian frequencies. PIP5k1β^−/−^ mice had a similar body structure to that of wild-type (WT) littermates, with no obvious differences observed between PIP5k1β^−/−^ and WT controls (Supplementary Figure S1A). Interestingly, micro-computed tomography (micro-CT) analysis revealed that the trabecular bone mineral density (BMD), trabecular bone volume vs. tissue volume (BV/TV), trabecular bone number (Tb.N.), and trabecular bone thickness (Tb.Th.) were significantly lower, but that trabecular bone separation (Tb.Sp.) was higher in PIP5k1β^−/−^ mice compared to that of age- and sex-matched WT littermates (Figure 1A–F), suggesting defects in trabecular bone growth. In comparison, there were no significant differences in cortical bone parameters, such as cortical bone mass, cortical BMD, and cortical bone thickness, between WT and PIP5k1β^−/−^ mice (Supplementary Figure S2). To gain further insight into the *in vivo* cellular phenotype of the PIP5k1β^−/−^ mice, bone histomorphometry was performed on decalcified sections stained for tartrate-resistant acid phosphatase (TRAP) activity and for hematoxylin and eosin (H&E) staining (Figure 1G). Consistent with micro-CT data, histomorphometric analysis of tibia from 8-week-old PIP5k1β^−/−^ mice revealed a lower trabecular bone mass when compared to WT mice (Figure 1G and H). Analysis of osteoclast parameters using TRAP stained sections showed that PIP5k1β^−/−^ mice exhibited a significant increase in the number of osteoclasts (Figure 1G and I). Moreover, dynamic histomorphometric analysis detected a reduced bone formation rate and mineral apposition rate in PIP5k1β^−/−^ mice (Figure 1J and K). Furthermore, the serum levels of serum type I collagen cross-linked c-terminal peptide 1 (CTX-1), a marker for bone resorption, were significantly increased (Figure 1L), but serum osteocalcin (OCN) levels, a marker for bone formation, were dramatically decreased (Figure 1O) in PIP5k1β^−/−^ mice. Immunohistochemistry detected that expression of Cathepsin K (CTSK), one of the major biomarkers of mature osteoclasts, was significantly higher in PIP5k1β deletion mice than in wide-type mice (Figure 1M and N). Collectively, these results suggest that PIP5k1β deletion leads to a significant decrease in bone mass, possibly due to effects on osteoclastic bone resorption and osteoblastic bone formation.

### PIP5k1β expression was increased during RANKL-induced osteoclast differentiation

To determine how PIP5k1β influences osteoclast formation and function, we first examined the transcriptomes of pre-osteoclasts and mature osteoclasts differentiated from mouse BMMs using cDNA microarray and found that among PIP5k1s, only PIP5k1β was highly expressed in mature osteoclasts (Figure 2A). When we cultured BMMs in the presence of RANKL and macrophage colony-stimulating factor (M-CSF), the expression of PIP5k1β was markedly increased during RANKL-induced osteoclast differentiation from the third day of RANKL induction (Figure 2B); however, the expressions of PIP5k1α and PIP5k1γ were not changed (Figure 3B). Expression levels of v-ATPase-d2 and TRAP, important markers of osteoclastogenesis, increased as the cells differentiated (Figure 2B). To confirm the importance of PIP5k1β in skeletal biology, we explored the expression pattern of PIP5k1β in different mouse organs such as bone, spleen, brain, thymus, lung, kidney, liver, heart, and muscle, and found that PIP5k1β was highly expressed in heart, lung, brain, and bone (Supplementary Figure S1B), which indicated that PIP5k1β might play a crucial role in bone biology. These results indicate that PIP5k1β might be involved in regulation of osteoclastogenesis and bone remodeling.

**Figure 4:**
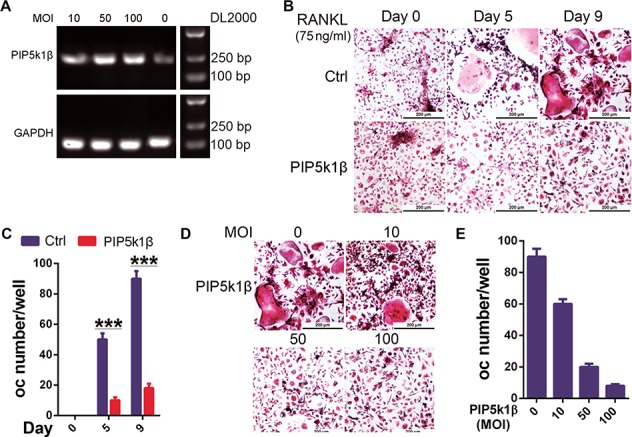
Overexpression of PIP5k1β arrests osteoclast formation. (**A**) WT BMMs were transduced with LV5-NC or gradually increasing concentration of LV5-PIP5k1β, and the expression of PIP5k1β was detected by RT-PCR. (**B** and **C**) BMMs transduced with LV5-NC or 50 MOI of LV5-PIP5k1β were cultured with RANKL and M-CSF for 0, 5, or 9 days. The cells were stained for TRAP activity and the number of osteoclasts (oc) was counted. (**D** and **E**) BMMs from **A** were cultured with RANKL and M-CSF for 9 days. The cells were stained for TRAP activity and oc number was counted. Scale bar, 200 μm. All the experiments were repeated three times. Data represent mean ± SD of three independent tests. ***P < 0.001.

**Figure 5:**
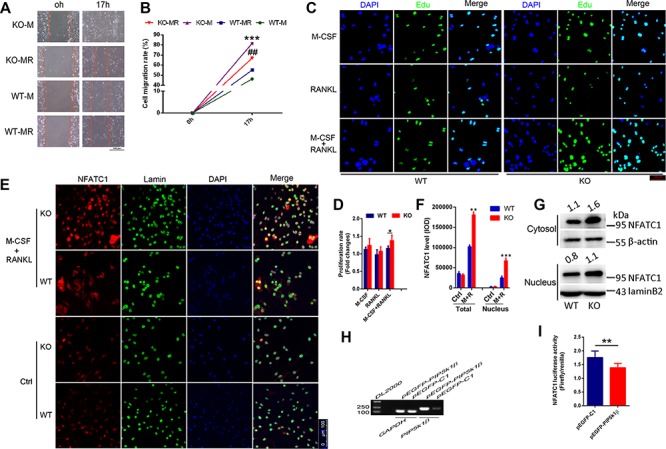
Deficiency of PIP5k1β enhances proliferation, migration, and NFATC1 nuclear localization in pre-osteoclasts. (**A** and **B**) Wound healing assay of WT or PIP5k1β^−/−^ BMMs with M-CSF or both M-CSF and RANKL. (**A**) Cells migrating to the scratches were monitored and images were taken at 0 and 17 h after wounding. (**B**) The migration rate was calculated. (**C** and **D**) Edu incorporation assay (**C**) and CCK8 assay (**D**) were performed to test the proliferation rate of WT or PIP5k1β^−/−^ BMMs under treatment with M-CSF, RANKL, or both. (**E**–**G**) Nuclear translocation of NFATC1 in WT or PIP5k1β^−/−^ osteoclasts stimulated with M-CSF and RANKL was detected by immunofluorescence (**E**) and western blotting (**G**). (**F**) The nuclear translocation in **E** was quantified using Image-Pro Plus Microsoft. (**H** and **I**) RAW264.7 cells stably transfected with an NFATC1 luciferase reporter construct were transduced with vector or pEGFP-PIP5k1β for 24 h, followed by incubation with RANKL (100 ng/ml) for 4 h. NFATC1 luciferase activity was measured (**I**) and PIP5k1β mRNA levels were detected via RT-PCR (**H**). All experiments were repeated three times. Data represent mean ± SD of triplicate tests. *P < 0.01; **P < 0.001; ^##^*P* < 0.01; ***P < 0.0001 vs. WT.

### PIP5k1β-deficient osteoclasts exhibit normal morphology

Accumulating evidence suggests that PIP5k1 family kinases serve central roles in cytoskeleton assembly (Shibasaki et al., 1997; Rozelle et al., 2000; van Horck et al., 2002; Kisseleva et al., 2005; Wang et al., 2008b; van den Bout and Divecha, 2009), so we formulated the hypothesis that PIP5k1β deficiency may affect the osteoclast cytoskeleton. To verify this hypothesis, we would examine actin ring formation as well as the localization of vinculin and Rac1, which are critical factors that can contribute to the ability of osteoclasts to rearrange podosomes into the sealing zone and establish the bone-resorbing apparatus, through classical confocal microscopy (Ory et al., 2000; Sun et al., 2005; Wang et al., 2008a; Croke et al., 2011; Fukunaga et al., 2014). F-actin exhibits a similar well-defined peripheral belt architecture in both WT and PIP5k1β-deficient osteoclasts, with PIP5k1β deletion promoting podosome formation in pre-osteoclasts and the formation of much larger actin rings in mature osteoclasts relative to WT controls. Vinculin and Rac1 localization and expression are also normal in PIP5k1β-deficient osteoclasts, which are found to be enriched at the podosome belt and are colocalized with F-actin (Touaitahuata et al., 2016) (Figure 2C). Finally, the expression level of β3 integrin, which is involved in the association of osteoclasts with the bone matrix to trigger osteoclast differentiation and bone resorption activity, was also unaffected by PIP5k1β deficiency (Supplementary Figure S1C and D).These results indicate that PIP5k1β deletion accelerates actin ring formation but does not affect osteoclast cytoskeleton assembly, sealing zone formation or ability to interact with the bone matrix.

**Figure 6:**
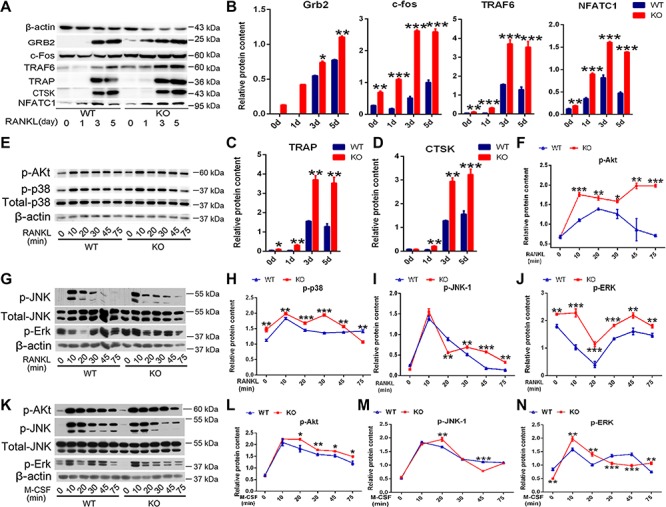
Deficiency of PIP5k1β leads to increases in M-CSF- and RANKL-mediated MAPK, c-Fos, and AKT signaling cascades. (**A**–**D**) WT or PIP5k1β^−/−^ BMMs were cultured with M-CSF (20 ng/ml) and RANKL (75 ng/ml) for 0, 1, 3, 5 days. (**A**) Expression of TRAF6, c-Fos, Grb2, TRAP, and CTSK were analyzed by western blotting. Actin served as loading controls. (**B**–**D**) The band densitometry was quantified and normalized to β-actin using ImageJ. (**E**–**J**) Serum- and cytokine-starved WT and PIP5k1β^−/−^ macrophages were exposed to RANKL (100 ng/ml). phosphorylated AKT, JNK, ERK, and p38 were immunoblotted. Actin served as loading controls, and the expression levels were analyzed by ImageJ. (**K**–**N**) Serum- and cytokine-starved WT and PIP5k1β^−/−^ macrophages were exposed to M-CSF (30 ng/ml). Phosphorylated AKT, ERK, and JNK were immunoblotted. Actin served as loading control, and the expression levels were analyzed by ImageJ. All experiments were repeated three times. Data represent mean ± SD. *P < 0.05; **P < 0.01; ***P < 0.001 vs. WT.

**Figure 7:**
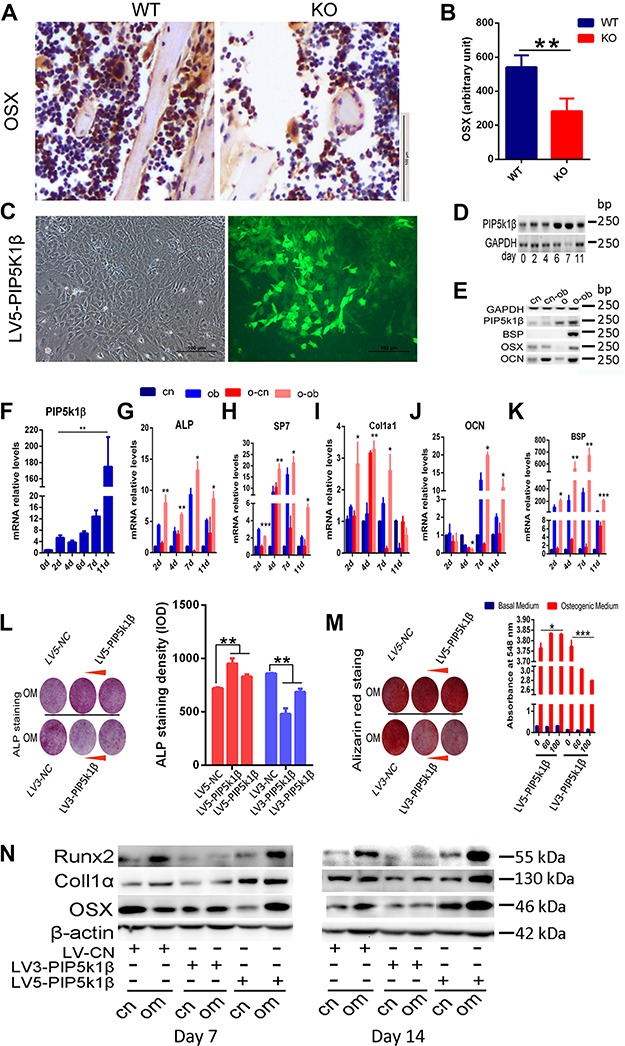
PIP5k1β accelerates osteoblast differentiation. (**A** and **B**) The expression of OSX in WT or PIP5k1β knockout mice was determined by immunohistochemistry (**A**) and the staining density was quantified by ImageJ (**B**). *n* = 6 per group. (**C**) WT BMSCs were transfected with vectors or LV3-PIP5k1β (knockdown) or LV5-PIP5k1β (overexpression). The transfection ratios of LV5-PIP5k1β are shown. (**D**–**K**) BMSCs from **C** were cultured with osteogenic medium for the indicated times. RT-PCR was performed to determine PIP5k1β, BSP, OSX, and OCN expression levels (**D** and **E**) and Q-PCR was performed for the mRNA expression of PIP5k1β, Alpl (ALP), Ibsp (BSP), SP7, OCN, and Col1α1 (**F**–**K**). Data present mean ± SD of triplicate samples. cn, control cells treated with control medium; o and o-cn, cells overexpressing PIP5k1β treated with control medium; cn-ob, control cells treated with osteogenic medium; o-ob, cells overexpressing PIP5k1β treated with osteogenic medium. (**L**) Cells from **C** cultured for 7 days with osteogenic medium were fixed and stained for ALP. (**M**) Cells cultured with osteogenic medium for 14 days were fixed and stained for Alizarin red and quantified by densitometry at 548 nm. (**N**) Western blot for osteoblast marker genes after 7 and 14 days of treatment with osteogenic medium. β-actin was used as loading control. All experiments were performed in triplicate. Data represent mean ± SD of triplicate samples. *P < 0.05; **P < 0.01; ***P < 0.001 vs. control. cn, cells treated with control medium; om, cells treated with osteogenic medium.

### Deficiency of PIP5k1β enhanced osteoclast differentiation and bone resorption

Given our observation that PIP5k1β expression is upregulated during RANKL-induced osteoclastogenesis, we examined the role of PIP5k1β in osteoclast differentiation. Bone marrow cells from 9-week-old WT and PIP5k1β^−/−^ mice were cultured with M-CSF to generate WT and PIP5k1β^−/−^ BMMs, which were used as osteoclast precursor cells to be stimulated with RANKL and M-CSF. Deletion of PIP5k1β in BMMs significantly stimulates the formation of TRAP-positive multinucleated cells mediated by RANKL (Figure 3A and C). More osteoclasts with 3–8 nuclei or >13 nuclei were observed in the PIP5k1β-deficient group relative to WT controls. Reverse transcription polymerase chain reaction (RT-PCR) results demonstrated an increased expression of dendritic cell-specific transmembrane protein (DC-STAMP) in PIP5k1β^−/−^ BMMs compared with WT BMMs during RANKL-induced osteoclast differentiation (Figure 3B). Gene expression analysis revealed that PIP5k1β expression was significantly increased from the third day of RANKL-induced osteoclastogenesis, but that the expression of the other two PIP5k1 family proteins PIP5k1α and PIP5k1γ did not change significantly in WT or PIP5k1β deletion BMMs during osteoclastogenesis (Figure 3B and D). The expression of osteoclast marker genes, including TRAP, CTSK, v-ATPase-d2, NFATC1, DC-STAMP, and OSCAR, was dramatically enhanced during osteoclastogenesis of PIP5k1β^−/−^ BMMs relative to WT BMMs (Figure 3E–J). Furthermore, we cultured WT and PIP5k1β^−/−^ BMMs on bovine bone and initiated osteoclastogenesis by M-CSF and RANKL stimulation. After successful formation of mature osteoclasts, the cells were cultured for another day with M-CSF and RANKL stimulation to further resorb bone; the resorbed bones were then subjected to scanning electron microscopy (SEM). We found that PIP5k1β deletion promoted osteoclastic bone resorption, with the area of bone resorbed by the PIP5k1β^−/−^ osteoclasts being significantly larger than that by the WT osteoclasts (Figure 3K and L). These results suggest that PIP5k1β deficiency enhances osteoclast differentiation and formation as well as promoting osteoclastic bone resorption, and this function might not depend on a compensational role for PIP5k1α and PIP5k1γ.

**Figure 8:**
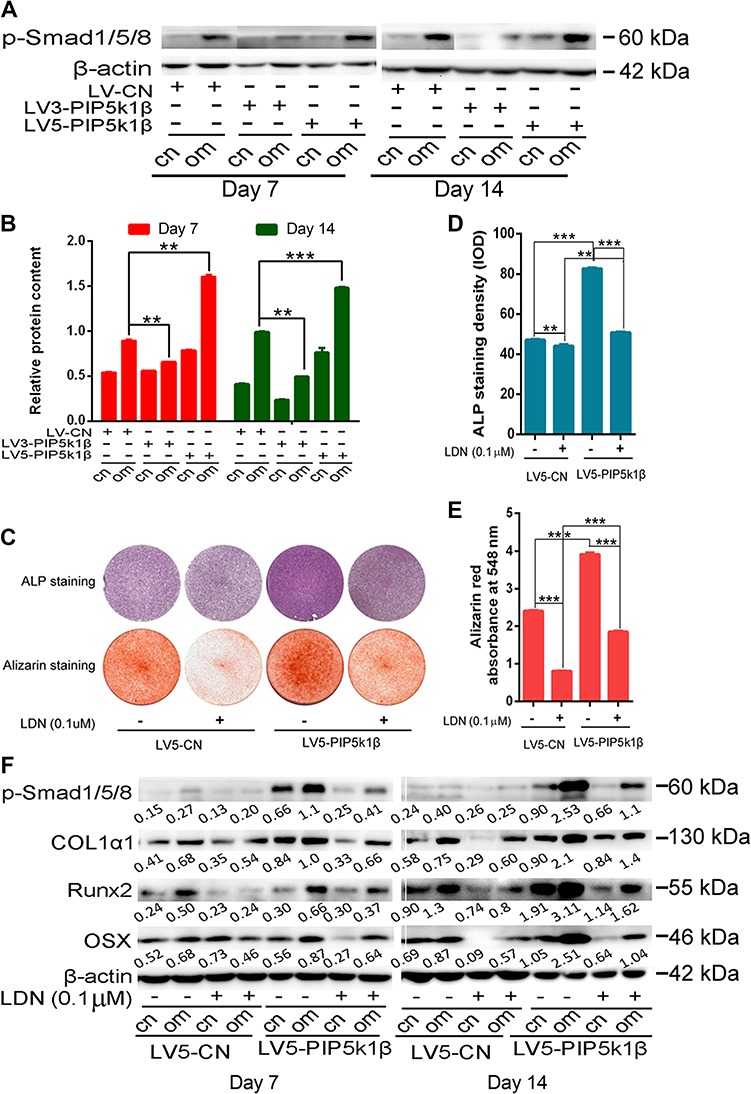
PIP5k1β accelerates osteoblast differentiation through activating p-Smad1/5/8 signaling. WT BMSCs were transfected with LV3-PIP5k1β (knockdown) or LV5-PIP5k1β (overexpression) and then incubated with control or osteogenic medium for 7 or 14 days. (**A**) Western blot for p-smad1/5/8 after 7 and 14 days of treatment with osteogenic medium. β-actin was used as loading control. (**B**) The band densitometry from **A** was analyzed with ImageJ. (**C**) ALP and Alizarin red S staining for BMSCs incubated with control or osteogenic medium with or without smad1/5/8-specific inhibitor LDN193189 for 7 and 14 days, respectively. (**D**) The ALP staining density was analyzed using the software Image-Pro Plus. (**E**) The calcium precipitates in **B** were dissolved in 0.1 N sodium hydroxide and quantified by absorbance at 548 nm with a Tecan Safire2 microplate reader. (**F**) Western blotting of BMSCs incubated with control or osteogenic medium with or without smad1/5/8-specific inhibitor LDN193189 for 7 and 14 days, respectively. Expression of p-smad1/5/8 and osteogenic marker genes was detected and analyzed with ImageJ. All experiments were performed in triplicate. Data represent mean ± SD of triplicate samples. *P < 0.05; **P < 0.01; ***P < 0.001 vs. control. cn, cells treated with control medium; om, cells treated with osteogenic medium.

### Overexpression of PIP5k1β arrests osteoclastogenesis

To further verify the role of PIP5k1β in osteoclast differentiation, we overexpressed PIP5k1β in BMMs and evaluated the effect on RANKL-induced osteoclast differentiation. PIP5k1β was overexpressed in the BMMs by lentivirus transfection into the BMM cells, with the BMMs subsequently cultured with RANKL and M-CSF. The increasing degree of PIP5k1β overexpression with gradually increased doses of lentivirus was demonstrated by RT-PCR (Figure 4A). TRAP staining analysis showed that overexpression of PIP5k1β significantly inhibits osteoclast differentiation, and that the inhibitory effect gradually increased with increasing magnitude of PIP5k1β overexpression (Figure 4B–E).

### Deficiency of PIP5k1β enhances proliferation, migration, and NFATC1 nuclear localization of pre-osteoclasts

To explore the mechanisms of PIP5k1β inhibition on osteoclast differentiation, we examined the effect of PIP5k1β on proliferation and migration of BMMs. The wound healing assay displayed that PIP5k1β deletion promotes migration of pre-osteoclasts triggered not only by M-CSF or by both M-CSF and RANKL (Figure 5A and B). M-CSF has been demonstrated to stimulate osteoclast precursors and pre-osteoclasts migration (Felix et al., 1994; Ross and Teitelbaum, 2005; Kim et al., 2016); however, the role of RANKL in osteoclastic cell migration is remaining to be explored. In Figure 5A and B, M-CSF alone exerts stronger effect on the migration of PIP5k1β knockout BMMs than M-CSF and RANK together do; this might be attributed to when under PIP5k1β deletion and RANKL stimulation, BMMs osteoclast differentiation ability was much increased, and the osteoclast reorganize and maturation may influence cells migration. The Edu incorporation assay and CCK8 assay showed that PIP5k1β deficiency enhanced proliferation of BMMs under the induction of RANKL and M-CSF, whereas treatment with either RANKL or M-CSF alone had no significant effect on the proliferation of the BMMs (Figure 5C and D). Furthermore, PIP5k1β deficiency facilitated NFATC1 expression and nuclear translocalization when the cells were cultured with RANKL, as detected by classical confocal microscopy and western blotting (Figure 5E–G). A luciferase assay conducted with an NFATC1 responsive reporter was found to be strongly dampened by PIP5k1β overexpression under stimulation with RANKL (Figure 5H and I). These results imply that PIP5k1β suppresses osteoclast differentiation by depressing pre-osteoclast proliferation, migration, and also reducing NFATC1 signaling by decreasing its expression and nuclear translocalization.

### Deficiency of PIP5k1β leads to increased M-CSF- and RANKL-mediated MAPK, c-Fos, and AKT signaling cascades

PIP5k1β was identified as highly expressed during RANKL-mediated osteoclastogenesis, and PIP5k1β suppressed osteoclast formation and bone resorption capabilities. We asked which signaling pathways modulate osteoclast differentiation and are affected by the absence of PIP5k1β, which in turn influences osteoclastogenesis. First, we examined the established RANKL signaling mediators, such as c-Fos, TRAF6, NFATC1, CTSK, and TRAP, and found that deletion of PIP5k1β prominently enhanced the expression of these genes during RANKL-mediated osteoclastogenesis (Figures 3E, F, H, and 6A–D). During RANKL-induced osteoclastogenesis, ligation of RANKL with its receptor RANK leads to recruitment of TRAF6 by RANK, which leads to activation of NF-κB, MAPK, and Akt pathways (Kobayashi et al., 2001; Kadono et al., 2005). In this context, we examined which of these pathways are modulated by PIP5k1β. The RANKL-induced activating phosphorylation of Akt and three typical MAP kinases (p38, ERK1/2 and JNK) were substantially elevated in PIP5k1β deletion BMMs compared with that of WT BMMs (Figure 6E–J).

We then treated WT and PIP5k1β^−/−^ mice osteoclast precursors with M-CSF and assessed activation of established effector molecules, which mediate osteoclast formation. Absence of PIP5k1β had certain stimulatory effects on M-CSF-induced phosphorylation of ERK, JNK, or AKT, but the enhanced effects were not consistent with the exposure time of the cells to M-CSF, especially for JNK and ERK1/2, compared with WT BMMs (Figure 6K–N). Notably, the expression of Grb2 was also enhanced during RANKL-mediated osteoclastogenesis in PIP5k1β^−/−^ osteoclasts compared with WT osteoclasts (Figure 6A).The adaptor protein Grb2 has been reported to play a pivotal role in several tyrosine kinase signal transduction pathways, and a previous study demonstrated that Grb2 promotes osteoclast survival through Erk activation and bone resorption activity by enhancing their adhesion to bone (Levy-Apter et al., 2014). Moreover, to verify that PIP5k1β deficiency influenced osteoclast differentiation through MAPK pathway, we administrated WT and PIP5k1β^−/−^ cells with specific inhibitors for MAPK/p38 (SB203580), MAPK/ERK (U0126-EtOH), JNK (SP600125), or these three inhibitors together during osteoclastogenesis induced by M-CSF and RANKL. The TRAP staining results showed that inhibitors for MAPK/p38, MAPK/ERK, and JNK significantly reduced TRAP positive cell numbers; however, PIP5k1β deficiency partly rescued these reductions (Supplementary Figure S3A and B). These data suggest that PIP5k1β deficiency influences M-CSF or RANKL-induced osteoclastogenic properties to a certain extent, particularly RANKL-induced c-Fos, MAPK, and AKT signaling pathways. Therefore, PIP5k1β-mediated suppression of osteoclast differentiation and function may occur through influencing M-CSF- and RANKL-mediated MAPK, c-Fos, and AKT signaling cascades.

### PIP5k1β accelerates osteoblast differentiation

Given that the serum bone formation marker OCN was significantly decreased in PIP5k1β^−/−^ mice compared with WT mice, we explored the effect of PIP5k1β on osteoblast differentiation and function to investigate the role of PIP5k1β in bone homeostasis. Immunohistochemistry revealed that expression of osterix (OSX), one of the major transcription factors that controls osteoblast differentiation, was notably lower in PIP5k1β deletion mice than in wide-type mice (Figure 7A and B). Bone mesenchymal stem cells (BMSCs) constantly overexpressing or with knockdown of PIP5k1β were then obtained by corresponding lentivirus transfection (Figure 7C) and underwent osteoblast differentiation by stimulation with osteogenic medium. MTT assay revealed that knockdown or overexpression of PIP5k1β has no effect on BMSCs proliferation (Supplementary Figure S4A). Q-PCR was performed to determine whether osteoblast marker genes were differentially expressed between WT and PIP5k1β overexpression osteoblasts. The mRNA levels of PIP5k1β were significantly enhanced during osteoblast differentiation, and the mRNA expression of the osteoblast marker genes, alkaline phosphatase (ALP), OSX, type I collagen α1 (Col1α1), OCN, and bone sialoprotein (BSP), were increased by overexpression of PIP5k1β (Figure 7D–K). Increased ALP activity was observed in PIP5k1β-overexpression osteoblasts on Day 7 of culture, and decreased ALP activity was observed in PIP5k1β-knockdown osteoblasts on Day 7 of culture as compared to WT osteoblasts by ALP staining (Figure 7L). The bone mineralization activity of osteoblasts derived from BMSCs constantly overexpressing or with knockdown of PIP5k1β also varied concordantly with ALP activity relative to WT osteoblasts, as determined by Alizarin red S staining and analysis on Day 14 of culture (Figure 7M). Furthermore, the protein levels of Col1α1, Runt-related transcription factor 2 (RUNX2), and OSX, which are marker genes of osteoblastogenesis, were all enhanced via PIP5k1β overexpression and decreased when PIP5k1β was knocked down (Figure 7N; Supplementary Figure S4B and C). These results imply that PIP5k1β enhances the differentiation of MSCs into osteoblasts.

### PIP5k1β enhances osteoblast differentiation through activating p-Smad1/5/8 signaling

Several signaling pathways are involved in regulation of BMSC differentiation into osteoblasts, of which smad1/5/8 signaling exerts a crucial role in osteogenesis. To understand through which pathways PIP5k1β modulates osteoblast differentiation, we tested the activation of the main pathways that regulate differentiation of MSCs into osteoblasts, including smad1/5/8 and β-catenin, and found that PIP5k1β prominently modulates smad1/5/8 activation. The phosphorylation of smad1/5/8 was significantly increased after 7 and 14 days of treatment with osteogenic medium compared with cells treated with control medium, while knockdown of PIP5k1β drastically depressed smad1/5/8 phosphorylation but overexpression of PIP5k1β outstandingly increased its phosphorylation (Figure 8A and B). To refine this finding, we further treated BMSCs with LDN193189, a smad1/5/8 specific inhibitor, to eliminate the activation of smad1/5/8. We observed that PIP5k1β overexpression prominently enhanced osteoblast differentiation and LDN193189 significantly reduced osteogenesis, but the reduction ratio was partly rescued by overexpression of PIP5k1β (Figure 8C–E). Western blotting showed that PIP5k1β overexpression significantly enhanced expression of the osteoblastogenesis master genes Col1α1, Runx2, and OSX, and LDN193189 significantly inhibited expression of these genes, but the inhibition ratio was partly rescued by overexpression of PIP5k1β. Meanwhile, phosphorylation of smad1/5/8 varied in a consistent manner with expression of these osteoblastogenesis master genes (Figure 8F). In summary, these observations indicate that PIP5k1β enhances differentiation of BMSC into osteoblasts, partly through activating smad1/5/8 signaling.

## Discussion

Several therapeutic strategies have been developed for excessive bone loss diseases such as osteoporosis, including direct suppression of osteoclast resorption capacity or stimulation of osteoblast bone formation, with compromised efficacy and significant side effects. Therefore, more appropriate treatments need to be explored to help synchronize the action of osteoblasts and osteoclasts. In present study, we discovered that PIP5k1β inhibited osteoclast formation and function while facilitating osteoblast differentiation, so that PIP5k1β^−/−^ displayed an obvious osteoporosis phenotype. This suggests that PIP5k1β may serve a pivotal role in maintaining bone homeostasis and that investigation of the mechanisms behind its function may provide new strategies for the prevention and treatment of osteoporosis. Interestingly, genetic variation within the human *PIP5k1B* gene has been identified as suggestively associated with pit-and-fissure dental caries in humans, a phenotype that has been shown to correlate with BMD (Fabiani et al., 2006; Zeng et al., 2013).

Among all the family members of PIP5k1 family kinases that generate PIP2, only PIP5k1β was highly expressed during osteoblastogenesis and RANKL-induced osteoclast differentiation, which indicates that PIP5k1β might serve crucial roles in bone biology. Considering that PIP5k1 family proteins are reported to be involved in cytoskeleton assembly and that podosomes reorganization within osteoclasts is crucial for their formation and activity (Mao and Yin, 2007; van den Bout and Divecha, 2009; Teitelbaum, 2011; Touaitahuata et al., 2014), we speculated that PIP5k1β might participate in osteoclast sealing zone organization. However, our present study showed that PIP5k1β deletion osteoclasts exhibit no apparent cytoskeletal abnormalities, with no obvious impact on the expression and colocalization of proteins involved in sealing zone formation, such as vinculin, Rac1, and F-actin. Additionally, the expression level of β3 integrin, which takes part in the association of osteoclasts with bone matrix to trigger bone resorption, was also uninfluenced by PIP5k1β deficiency (Supplementary Figure S1C and D). Collectively, this indicates that PIP5k1β does not affect osteoclast cytoskeleton formation or interaction with bone matrix, though deletion of PIP5k1β accelerates and enlarges osteoclast actin ring formation. The bone resorption ability of osteoclasts is dependent on the formation of the actin-rich sealing zone, an actin ring made of densely packed podosomes, which delimits the zone of bone resorption (Touaitahuata et al., 2014). Podosomes are highly dynamic structures that reorganize during osteoclast maturation and activity, and mediate osteoclasts attachment and migration, so as to facilitate osteoclasts formation and enhance their bone resorption capacity. Larger actin ring formation enlarges the attachment area of osteoclasts to bone surface, which in turn enhance osteoclasts resorption capacity. PIP5k1β does seem to influence osteoclast formation and function, which might be buttressed by the following facts. First, PIP5k1β deletion enhanced the expression of osteoclast marker genes such as acid phosphatase 5 (ACP5), CTSK, DC-STAMP, c-Fos, and NFATC1. PIP5k1β deficiency facilitated osteoclast bone resorption as demonstrated by enhanced pit formation activity and highly elevated serum levels of the bone resorption marker CTX-1 in PIP5k1β deletion mice. Finally, knockdown and overexpression of PIP5k1β in BMMs accelerated and depressed osteoclast differentiation, respectively, as detected by TRAP staining.

Osteoclasts are generated from mononuclear hematopoietic myeloid lineage cells, which are derived in the bone marrow and are attracted to the blood stream by chemotactic factors such as sphingosine-1 phosphate (S1P) and stroma-derived factor-1 (SDF-1). These circulating precursors migrate to bone surfaces undergoing resorption by the action of chemokines and other factors that are modulated by M-CSF and RANKL at these sites, where they fuse to develop multinucleated bone-resorbing cells (Kikuta et al., 2011; Kular et al., 2012). Interestingly, in the present study, we demonstrated that PIP5k1β deletion can accelerate pre-osteoclast migration triggered by M-CSF or by both M-CSF and RANKL. Besides, PIP5k1β deletion also promoted pre-osteoclast proliferation. These results indicate that PIP5k1β represses pre-osteoclast proliferation and migration to inhibit osteoclastogenesis, but the molecular mechanisms remain to be explored.

Furthermore, our present study suggests that PIP5k1β affects osteoclast differentiation and function through modulation of NFATC1 that represents a master switch that modulates terminal differentiation of osteoclasts downstream of RANKL (Takayanagi et al., 2002; Asagiri et al., 2005), as supported by several discoveries. First, we found that PIP5k1β deficiency enhanced NFATC1 expression and facilitated NFATC1 nuclear translocation. Additionally, overexpression of PIP5k1β dampened NFATC1 activity. Moreover, the phosphorylation of Akt and the activation of MAPK/p38, ERK1/2, and JNK, which mediated NFATC1 activation during RANKL-stimulated osteoclast differentiation, were prolonged and enhanced with RANKL stimulation in PIP5k1β^−/−^ osteoclasts. Finally, PIP5k1β deletion enhanced TRAF6 and c-Fos expression, which were demonstrated to stimulate downstream mediators to trigger expression and nuclear translocalization of NFATC1 resulting in enhanced osteoclastogenesis (Wang et al., 1992; Kadono et al., 2005; Boyce and Xing, 2008). PIP5k1γ was reported to modulate calcium transport, which in turn, initiated NFATC1 (Wang et al., 2004). Moreover, NFATC1 can autoamplify and trigger calcitonin receptor expression that eventually upregulated calcium signaling (Asagiri et al., 2005). Further studies need to be performed to determine if PIP5k1β deletion affects calcium signaling.

The adaptor protein Grb2, which serves an important role in several tyrosine kinase signal transduction pathways, was reported to play a crucial role in osteoclastogenesis by increasing Erk and Akt signaling motivated by M-CSF to promote osteoclast proliferation, differentiation, and survival (Ross and Teitelbaum, 2005; Takayanagi, 2007; Levy-Apter et al., 2014). An interesting finding in the present study was that Grb2 expression was dramatically upregulated during osteoclastogenesis when PIP5k1β was deleted, with enhanced ERK1/2 signaling also observed. This implies that PIP5k1β might modulate Grb2 expression to regulate osteoclastogenesis. However, further study is needed to clarify the association between PIP5k1β and Grb2 expression.

Our present work also discovered that PIP5k1β facilitated osteoblast differentiation. The immunohistochemistry results presented in Figure 7A showed that OSX^+^ cell numbers were prominently decreased both in bone marrow cells and in bone lining cells in PIP5k1β deletion mice compared with that of WT mice, which indicated that PIP5k1β might stimulate differentiation of MSCs into osteoblasts through upregulation of OSX expression. Furthermore, the expression of early and late osteoblast differentiation markers Col1α1, ALP, OCN, and BSP, as well as the osteoblast differentiation master transcription factors Runx2 and OSX, were all outstandingly elevated or reduced during osteoblastogenesis of PIP5k1β overexpression or knockdown BMSCs, respectively. Moreover, ALP and Alizarin red staining demonstrated that overexpression or knockdown of PIP5k1β promoted or repressed differentiation of MSCs into osteoblasts and calcium precipitation, respectively. Meanwhile, the phosphorylation of smad1/5/8 was also enhanced or dampened in accordance with overexpression or knockdown of PIP5k1β during BMSC differentiation into osteoblasts. Moreover, PIP5k1β overexpression can partly rescue the inhibitory effect on osteoblast differentiation and smad1/5/8 activation upon administration of LDN193189, a smad1/5/8 specific inhibitor. Collectively, this implies that PIP5k1β facilitates osteoblast differentiation from BMSCs partly through smad1/5/8 signaling.

In summary, our study explored the role of PIP5k1β in bone biology, particularly in osteoclast and osteoblast differentiation and function, for the first time. We found that PIP5k1β was highly expressed both during osteoclast and osteoblast differentiation and PIP5k1β can regulate bone mass and bone remodeling by inhibiting osteoclast differentiation and facilitating osteoblast differentiation (Supplementary Figure S4D). Further studies need to be performed to determine if these functions are partly caused by altered PIP2 levels as a result of PIP5k1β kinase activity during osteoclast and osteoblast differentiation. Further investigation of the RANKL modulation of PIP5k1β expression is needed to increase our understanding of the biological role of PIP5k1β in maintaining bone homeostasis and to explore new targets for prevention and treatment of bone disorders.

## Materials and methods

### Reagents

Alpha-minimum essential medium (MEM), fetal bovine serum, and penicillin were purchased from Gibco BRL. Recombinant mouse M-CSF was purchased from R&D Systems. Recombinant Murine sRANK Ligand was purchased from Peprotech. TRAP staining solution was obtained from Sigma-Aldrich. The Cell Counting Kit-8 (CCK-8) was obtained from Dojindo Molecular Technology. The Click-iT® EdU Imaging Kits (C10337) were purchased from Invitrogen Life Technologies.

### Animals

WT and PIP5k1β^−/−^ FVB mice were obtained from PBmice of Fudan University and housed five per cage under standard conditions (12 h light/12 h dark cycle, 21°C controlled temperature). Briefly, the DNA transposon *piggyBac (PB)* element carrying LINE 080318007-HRA/PB was inserted into mouse chromosome 19 and hit into the gene *PIP5k1β* according to the methods described previously (Ding et al., 2005; Sun et al., 2008).

### Sample preparation and skeletal morphology

For micro-CT analysis, right femurs of both WT and PIP5k1β^−/−^ mice were fixed with 4% paraformaldehyde (PFA) and analyzed by Scanco Medical CT-40 instruments. Trabecular bone analysis was performed on the secondary spongiosa region (300 μm below the growth plate with a total height of 1 mm toward the mid-shaft) of the distal femur. 3D images were generated in CTvol program (Skyscan). For histochemistry, proximal tibias from both WT and PIP5k1β^−/−^ mice were fixed in 4% PFA and embedded in paraffin, cut longitudinally into 4-μm-thick sections, and processed for H&E staining or TRAP staining after decalcifying. To evaluate bone formation rate *in vivo*, WT and PIP5k1β^−/−^ mice were injected with green fluorescent calcein (Sigma-Aldrich; 5 mg/kg body weight) at 8 and 2 days before euthanasia. Then the tibiae were dissected and embedded in methyl methacrylate resins. Tissue sections were observed under a laser-scanning microscope (LSM5 PASCAL; Carl Zeiss).

### Cell culture

WT or PIP5k1β^−/−^ BMMs were obtained as described previously (Qin et al., 2012; Li et al., 2013). In brief, bone marrow cells extracted from the femurs and tibiae of 9–10-week-old WT and littermate PIP5k1β^−/−^ mice were cultured in α-MEM medium containing 15 ng/ml M-CSF (416-ML; R&D) in a T-75 cm^2^ flask for proliferation for 2–3 days until reaching 90% confluence. The cells were washed with phosphate buffer solution (PBS) three times and trypsinized for 30 min. Adherent cells were classified as BMMs. WT and PIP5k1β^−/−^ BMMs were plated on 96-well plates at a density of 8 × 10^3^ cells/well in triplicate and incubated with α-MEM medium containing 20 ng/ml M-CSF in a humidified incubator containing 5% CO_2_ at 37°C for 2 days. The cells were then cultured with α-MEM medium with M-CSF (20 ng/ml), RANKL (75 ng /ml; 315-11; Peprotech), and indicated inhibitors for indicated times. The medium was changed every other day. The inhibitors for p38/MAPK (SB203580), MAPK/ERK (U0126-EtOH), and JNK (SP600125) were purchased from Selleckchem. For osteoblast differentiation, BMSCs were gained from 9–10-week-old mice and expanded as previously described, induced by culturing cells in osteogenic medium (DMEM containing 1 M β-glycerophosphate, 50 mM ascorbic acid, and 1 mM methylisobutylxanthine) for indicated times, followed by subsequent experiments.

### TRAP activity assay

After 7 days of culture, the osteoclasts were fixed with 4% PFA in PBS for 10 min and then rinsed three times with PBS, followed by TRAP staining, using an acid phosphatase kit (387A; Sigmae-Aldrich) according to the manufacturer’s description, and counter-stained with hematoxylin for 10–30 sec. Images were obtained with a Nikon SMZ 1500 stereoscopic zoom microscope (Nikon Instruments Inc.). We randomly quantified the total number of osteoclasts on five selected fields of view for each sample.

### Immunofluorescence

For F-actin ring immunofluorescent staining, WT and PIP5k1β^−/−^ osteoclasts were fixed with 4% PFA for 15 min at room temperature and permeabilized for 5 min with 0.1% (*v/v*) Triton X-100 on ice. Cells were incubated with fluorescent phalloidins (1:40; Invitrogen Life Technologies) diluted in 1% (*w/v*) bovine serum albumin-PBS for 20 min at room temperature and then washed extensively with PBS. Cells were then incubated with Hoechst 3342 dye (1:5000; Invitrogen Life Technologies) for visualizing nuclei, washed with PBS, and mounted with ProLong Gold anti-fade mounting medium (Invitrogen Life Technologies). Fluorescence was detected with NIKON A1Si spectral detector confocal system equipped with 20 (dry) lenses. Fluorescence images were obtained with NISeC Elements software (National Institutes of Health).

### Pit formation assay

For the bone resorption assay, 8 × 10^3^ cells/well WT or PIP5k1β^−/−^ BMMs were plated onto bovine bone slices in 96-well plates with three replicates and treated with M-CSF (20 ng/ml) and RANKL (75 ng/ml) for 8 days. The bone slices were then fixed with 2.5% glutaraldehyde, followed by visualization with a SEM (FEI Quanta 250). Pit areas were quantified with Image J software (National Institutes of Health). Similar independent experiments were repeated three times.

### Cell proliferation and migration assay

The proliferation rate was determined using CCK-8 assay and Edu incorporation assay according to the manufacturer’s protocols. WT or PIP5k1β^−/−^ BMMs were seeded in 96-well plates at a density of 8 × 10^3^ cells/well and glass-bottom cell culture dish specific for confocal imaging and incubated in complete α-MEM supplemented with 20 ng/ml M-CSF for 24 h until 60% confluent. Cells were treated with 20 ng/ml M-CSF or 75 ng/ml RANKL or both for 24 h. Then 100 μl CCK-8 buffer was added to each well, followed by incubation at 37°C for additional 2 h. The absorbance was then measured at a wavelength of 450 nm (650 nm reference) with an ELX800 absorbance microplate reader (Bio-Tek). The migration ability was monitored with a wound healing assay. In a typical procedure, a straight scratch was made gently through the cell monolayer using an Eppendorf tip in 48-well plates. Detached cells were washed away with PBS and serum-free culture medium was added; cells were treated with 20 ng/ml M-CSF or 75 ng/ml RANKL or both. Cells migrating to the scratch were monitored and images were taken at 0 and 17 h after wounding. The migration rate was calculated following the equation: percentage wound healing = [(wound length at 0 h) − (wound length at 17 h)] / (wound length at 0 h) × 100 (Davalos et al., 2012).

### RNA extraction and Q-PCR assay

WT and PIP5k1β^−/−^ BMMs were plated in 6-well plates at a density of 1 × 10^5^ cells/well and incubated in complete α-MEM supplemented with 20 ng/ml M-CSF and 75 ng/ml RANKL for indicated number of days. Total RNA was extracted with TRIzol reagent (Invitrogen) following the manufacturer’s instructions and cDNA was generated with 1 μg of RNA. Q-PCR was then performed via the LightCycler480 system (Roche) using SYBR1Premix Ex TaqTM (TaKaRa) following the manufacturer’s instructions and data were analyzed using the comparison Ct (2^−∆∆^Ct) method. The following thermocycler protocol was employed for Q-PCR: denaturation at 95°C for 10 sec, 40 cycles at 95°C for 10 sec, and 60°C for 30 sec. Dissociation curve analysis was performed at the end of the amplification procedure, which did not show any nonspecific amplification. Glyceraldehyde-3-phosphate dehydrogenase (GAPDH) was used as housekeeping gene. All reactions were run in triplicate. The mouse primers used in the present study are as follows: GAPDH forward 5′-TTCACCACCATGGAGAAGGC-3′ and reverse 5′-GGCATGGACTGTGGTCATGA-3′; NFATC1 forward 5′-TCCGAGAATCGAGATCACCT-3′ and reverse 5′-AGGGGTCTCTGTAGGCTTCC-3′; ACP5 forward 5′-CGTCTCTGCACAGATTGCAT-3′ and reverse 5′-AACTGCTTTTTGAGCCAGGA-3′; CTSK forward 5′-GGACCCATCTCTGTGTCCAT-3′ and reverse 5′-CCGAGCCAAGAGAGCATATC-3′; DC-STAMP forward 5′-ACAAACAGTTCCAAAGCTTGC-3′ and reverse 5′-TCCTTGGGTTCCTTGCTTC-3′; OSCAR forward 5′-CTGCTGGTAACGGATCAGCTCCCCAGA-3′ and reverse 5′-CCAAGGAGCCAGAACCTTCGAAACT-3′; PIP5k1β forward 5′-GAAGAAGCCCTGGGATCCCGACA-3′ and reverse 5′-GGGTGTTTGGCTCAGCCGTCA-3′; v-ATPase-d2 forward 5′-AGACCACGGACTATGGCAAC-3′ and reverse 5′-CAGTGGGTGACACTTGGCTA-3′.

### Western blotting

Cells were lysed on ice for 30 min with RIPA lysis buffer, which contains 50 mM Tris^_^HCl, pH 7.4, 150 mM NaCl, 1% Nonidet P-40, and 0.1% SDS supplemented with protease inhibitors (10 mg/ml leupeptin, 10 mg/ml pepstatin A, and 10 mg/ml aprotinin). For western blotting, 25 μg of protein sample was resolved on 12.5% SDS-PAGE and electrotransferred onto nitrocellulose membranes (Whatman). The primary antibodies used were as follows: phospho-Akt, total Akt, phospo-p44/p42 ERK, total p44/p42 ERK, phospho-p38, total-p38, phospo-JNK, and total-JNK were purchased from Cell Signaling Technology (1:1000 dilution ratio). Anti-NFATC1 antibody was purchased from Santa Cruz (1:500 dilution ratio). β-actin or GAPDH was used as loading control. Horseradish peroxidase-conjugated secondary antibodies were used at a 1:5000 dilution. The antigen–antibody complexes were visualized using the enhanced chemiluminescence detection system (Millipore) following the manufacturer’s instructions. Immunoreactive bands were quantitatively analyzed in triplicate by normalizing the band intensities to their respective controls on scanned films using ImageJ software.

### Alkaline phosphatase staining and Alizarin red staining

The cell layer was rinsed with PBS three times, followed by fixation in 4% PFA for 10 min at room temperature. Then cells underwent ALP or Alizarin red staining. ALP staining was performed according to the manufacturer’s instructions. The fixed cells were then incubated with buffer containing 0.1% naphthol AS-Bi phosphate (Sigma-Aldrich) and 2% fast violet B (Sigma-Aldrich). After incubation for 1 h at 37°C, the cell layer was washed with deionized water. For Alizarin red staining, the fixed cells were rinsed with double-distilled H_2_O (ddH_2_O) and then stained with 40 mM Alizarin red S (pH 4.9, Sigma) for 15 min with gentle agitation followed by washing five times with ddH_2_O. The calcium precipitates were then dissolved with 0.1 M sodium hydroxide and monitored using a Tecan Safire2 microplate reader (Tecan) by absorbance at 548 nm to quantify the degree of mineralization.

### Statistical analysis

The data are presented as mean ± SD (*n* is the number of tissue preparations, cells, or experimental replicates). For comparing groups of data, a two-tailed Student’s *t*-test was used. A value of *P* < 0.05 was considered to be statistically significant.

## Supplementary Material

Supplementary_material_mjz028

## Notes

**Author contributions**: X.Zhao and P.C. contributed to conception and design, manuscript writing, collection of data, data analysis, and interpretation; G.H. and C.W. contributed to collection of data and provision of study material; L.J. and J.Z. contributed to collection of data and manuscript revision; J.X. contributed to data analysis and manuscript revision; and X.Zhang contributed to conception and design, manuscript revision, and final approval of manuscript.
