# Supplementary material for: PIP5k1β controls bone homeostasis through modulating both osteoclast and osteoblast differentiation
Source: J Mol Cell Biol. 2019 Apr 16;12(1):55–70. doi: 10.1093/jmcb/mjz028 (PMC7052985; doi:10.1093/jmcb/mjz028)
Supplement: Supplementary_material_mjz028 [file supplementary_material_mjz028.pdf]

PIP5k1β controls bone homeostasis through modulating both osteoclast and osteoblast differentiation

Xiaoying Zhao[1, 2#], Penglei Cui[1#], Guoli Hu[1, 2],Chuandong Wang[1], Lei Jiang[4],Jingyu Zhao[1, 2], Jiake Xu[3], Xiaoling Zhang[1, 2*]

[1]Department of Orthopedic Surgery, Xinhua Hospital, School of Medicine, Shanghai Jiao Tong University, Shanghai 200092, China

[2] The Key Laboratory of Stem Cell Biology, Shanghai Institutes for Biological Sciences, Chinese Academy of Sciences, Shanghai 200025, China

[3]School of Pathology and Laboratory Medicine, University of Western Australia, Perth, Western Australia, 6009, Australia

[4]Key laboratory of Tibetan medicine research, Northwest Plateau Institute of Biology, Chinese Academy of Sciences, Xining 810001, PR China

[#] The two authors contributed equally to this work.

[*]Corresponding author: Department of Orthopedic Surgery, Xin Hua Hospital, School of Medicine, Shanghai Jiao Tong University (SJTUSM), Shanghai 200092, China. Tel: +86 21 54923338; Fax: +86 21 54923338; E-mail: xlzhang@shsmu.edu.cn

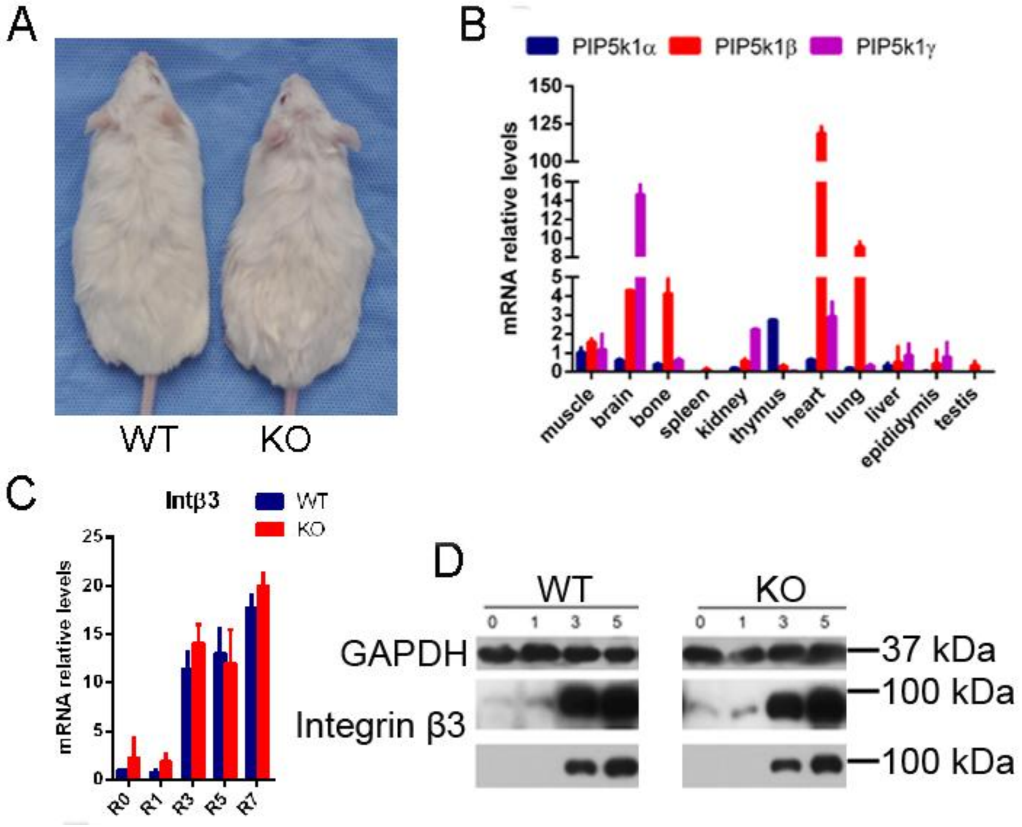

Supplementary Figure S1 (**A**) The whole body of WT and PIP5k1β deletion mice. (**B**) Total RNA was isolated from bone, spleen, brain, thymus, lung, kidney, liver, epididymis, testis, heart, and muscle of WT mice. Quantitative real-time PCR was performed for the mRNA expression of PIP5k1α, β and γ. (**C** and **D**) WT or PIP5k1$\beta^{-/-}$ BMMs were cultured with M-CSF (20 ng/ml) and RANKL (75 ng/ml) for 0, 1, 3, 5 or 7 days. β3 Integrin expression was analyzed by real-time polymerase chain reaction (Q-PCR) and the results were normalized to the expression of GAPDH (**C**) and western blotting (**D**). All experiments were performed at least three times; *$P < 0.05$; **$P < 0.01$.

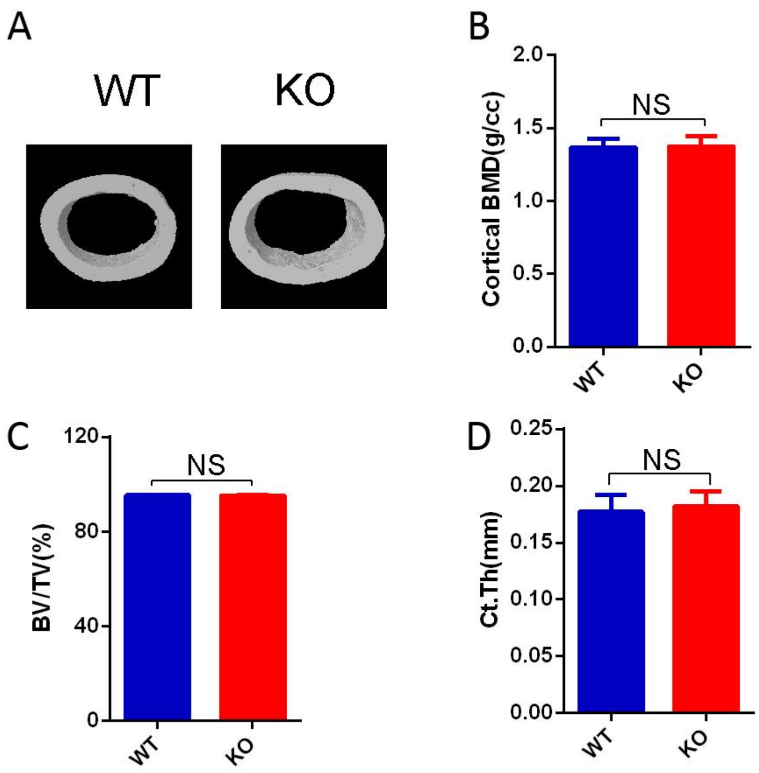

Supplementary Figure S2 PIP5k1β-deficient mice exhibit normal cortical bone phenotype. (**A**) Representative micro-CT images of cortical bone from femurs in WT or PIP5k1β knockout 2-month-old male mice. (**B**–**F**) Bone mineral density (BMD), Bone volume per tissue volume (BV/TV), cortical bone thickness (Ct.Th) were assessed from the micro-CT measurements (n = 10).

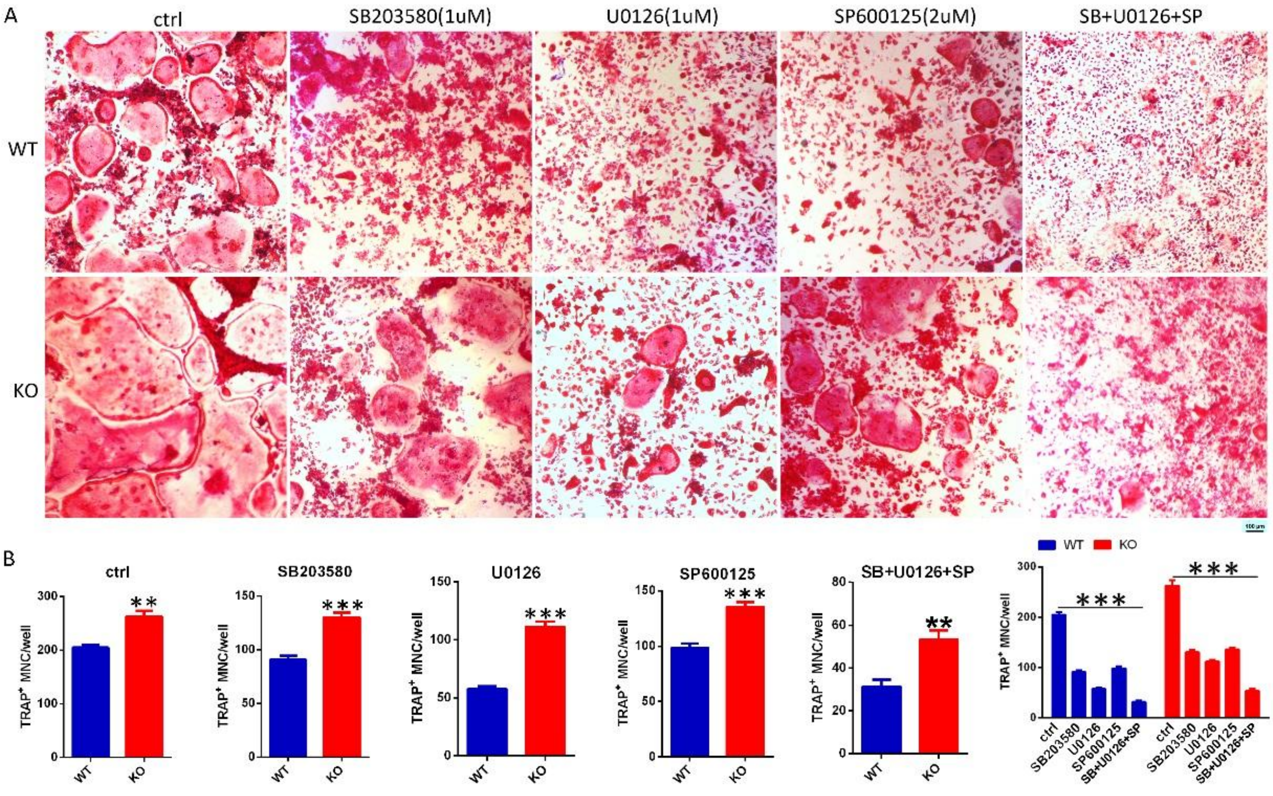

Supplementary Figure S3 PIP5k1β-deficiency partly rescued the inhibition of osteoclastogenesis by inhibitors specific for MAPK pathway. Bone marrow cells from WT or PIP5k1β$^{-/-}$ mice were treated with 15 ng/ml M-CSF for two days and the cells adhered to the cell plates were underwent osteoclastogenesis by stimulation with 20 ng/ml M-CSF and 75 ng/ml RANKL and inhibitors for MAPK/p38 (SB203580, 1 μM), ERK1/2 (U0126-EtOH, 1 μM), p-JNK (SP600125, 5 μM) or these three inhibitors together as indicated with indicated concentrations for the 5 days. The formation of osteoclasts was detected by TRAP staining (**A**) and the number of osteoclasts were counted (**B**). All experiments were performed triplicate. Data represent means ± SD of triplicate samples. ** $P < 0.01$; ***$P < 0.001$ versus WT.

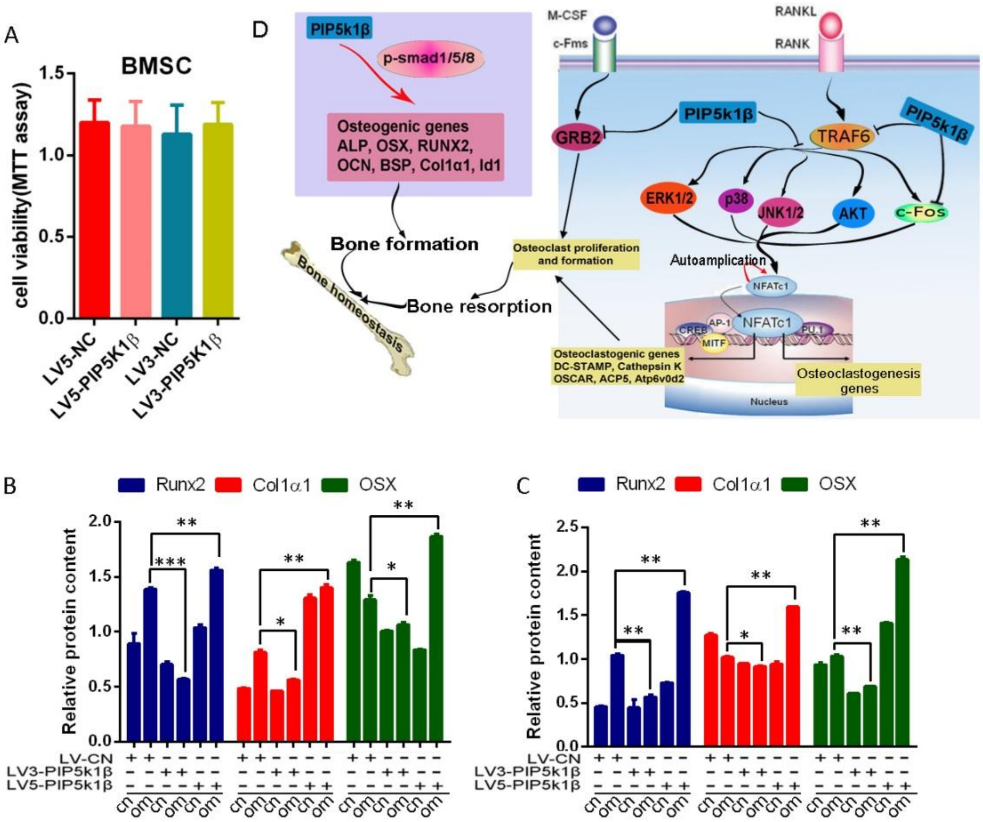

Supplementary Figure S4 (**A**) MTT assay to detect the proliferation rate of BMSCs constantly overexpressing or knockdown of PIP5k1β and the control BMSCs. (**B** and **C**) Densitometry analysis of Fig.7N and the results were shown graphically. All experiments were performed triplicate. Data represent means ± SD of triplicate samples. *P < 0.05; ** P < 0.01; ***P < 0.001 versus control; cn, cells treated with control medium; om, cells treated with osteogenic medium. (**D**) Model of PIP5k1β to modulate bone homeostasis. RANKL binds to RANK then RANK recruit TRAF6, which leads to phosphorylation of JNK, Akt, MAPK/Erk1/2 and MAPK/p38 signaling and activation of c-Fos, which are crucial for NFATC1 induction and activation. NFATC1 in turn initiates its own expression and activation. NFATC1 promotes osteoclastogenesis by promoting expression of osteoclastogenic genes such as DC-STAMP, Cathepsin k, OSCAR, ACP5 and Atpasev0d2. PIP5k1β inhibits RANKL-induced MAPK, AKT activation and TRAF6, c-Fos expression; PIP5k1β also inhibits Grb2 expression during osteoclastogenesis. Grb2 promotes osteoclast proliferation and formation. In this way, PIP5k1β inhibits osteoclastogenesis to modulate bone resorption. On the other hand, PIP5k1β promotes expression of osteogenic genes such as ALP, OSX, RUNX2, OCN, BSP and col1α1 to enhance bone formation through activating smad1/5/8 signaling.
